# Supplementary material for: An adaptive biomolecular condensation response is conserved across environmentally divergent species
Source: Nat Commun. 2024 Apr 11;15:3127. doi: 10.1038/s41467-024-47355-9 (PMC11009240; doi:10.1038/s41467-024-47355-9)
Supplement: Supplementary file 1 — Supplementary Information [file 41467_2024_47355_MOESM1_ESM.pdf]

Supplementary Figures

An adaptive biomolecular condensation response is conserved across environmentally divergent species

Samantha Keyport Kik, Dana Christopher, Hendrik Glauninger, Caitlin Wong Hickernell, Jared A. M. Bard, Kyle M. Lin, Allison H. Squires, Michael Ford, Tobin R. Sosnick, and D. Allan Drummond

Supplementary Figure S1.

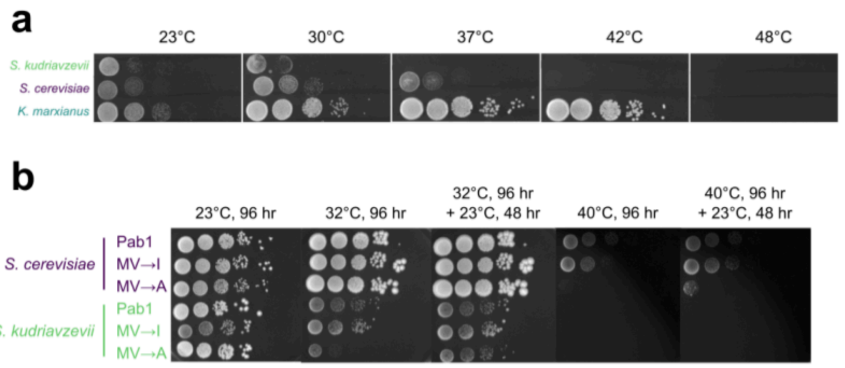

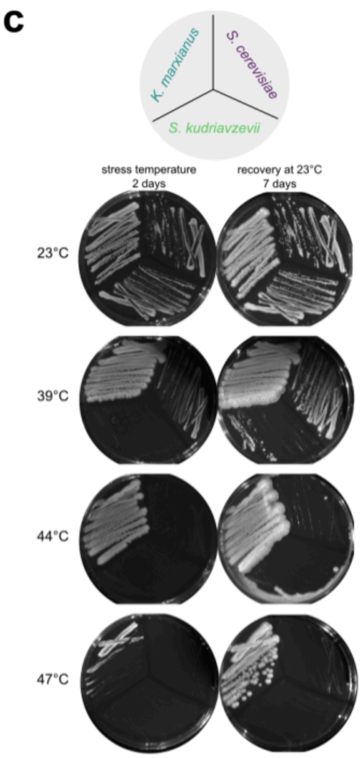

d

species	estimated T_{max} (°C) in culture	growth on plate at T_{max} + 1 °C?	growth on plate at T_{max} + 1 °C + recovery?
S. cerevisiae	43°C	✗	✓
S. kudriavzevii	38°C	✗	✓
K. marxianus	47°C	✓	✓

Figure S1. Growth, recovery, and death phenotypes of each three species. a Spot assays of *S. cerevisiae*, *S. kudriavzevii*, and *K. marxianus* strains. Plates were incubated at 23, 30, 37, 42, or 48°C for 2 days and then images. Columns are 10-fold dilutions. **b** Biological replicate of Figure 4e. **c** Growth on plates of each wild-type yeast species after a single-colony streak on a YPD plate. Each plate was incubated at either control or each species' T_{max} +1°C (calculated from culture) for two days, imaged, then shifted to room temperature, grown for 7 days, and imaged again. **d** Summary table of panel **c**.

Supplementary Figure S2.

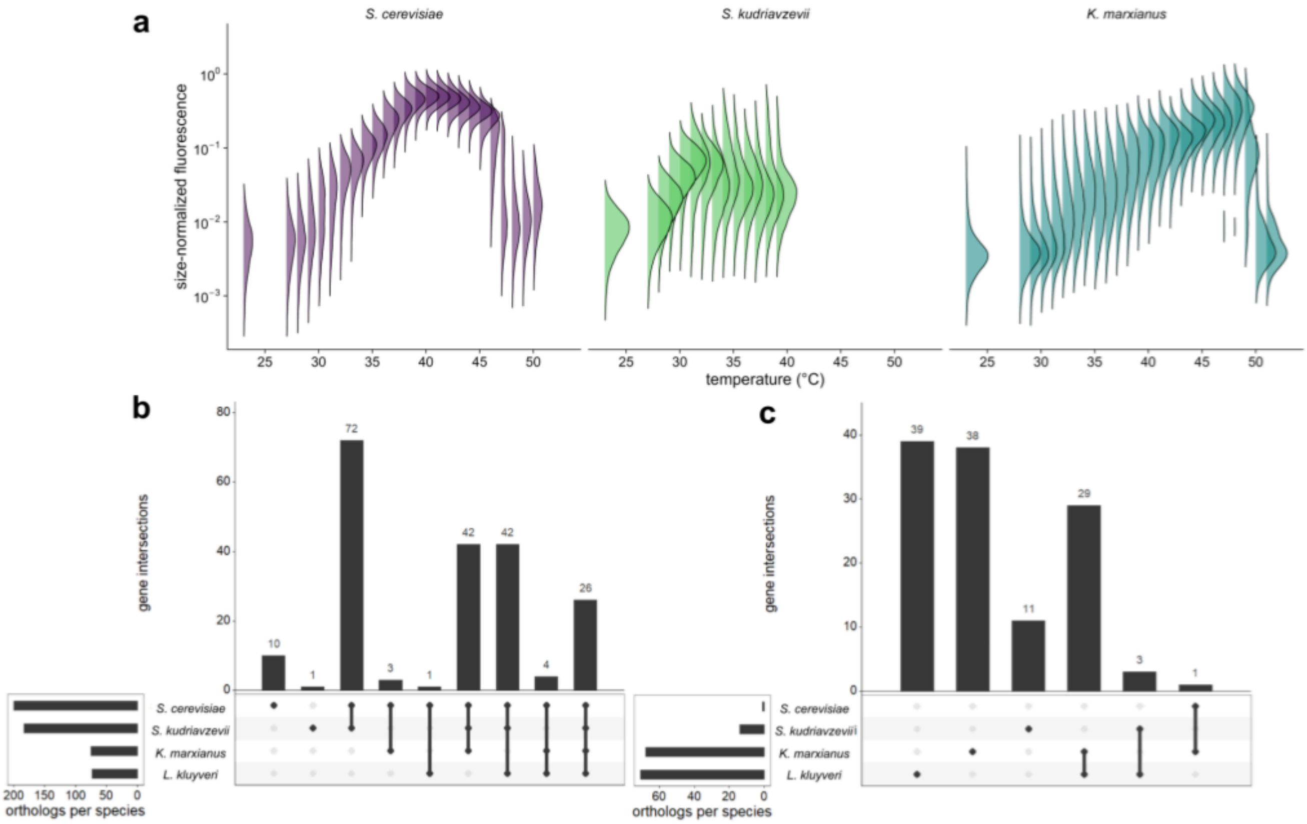

Figure S2. Expression dynamics across temperatures.
a Density distributions of red (endogenous SSA4-mCherry in *S. kudriavzevii* and *S. cerevisiae*) or green (plasmid-expressed SSA3p-eGFP in *K. marxianus*) fluorescence normalized by forward scatter for each species and temperature. Each distribution represents at least 5,000 cells. **b**, **c** comparison of Msn2/4 up (**b**) and downregulated (**c**) genes from our study and *L. kluyveri* from Brion et al., 2016. There is strong overlap in upregulated genes and lack of overlap in downregulated genes between pre- and post-duplication relatives, consistent with the hypothesis that the post-duplication orthologs should be substantially similar if there are some ancestral genes which were only recruited into the Msn2/4 post-whole-genome duplication.

Supplementary Figure S3.

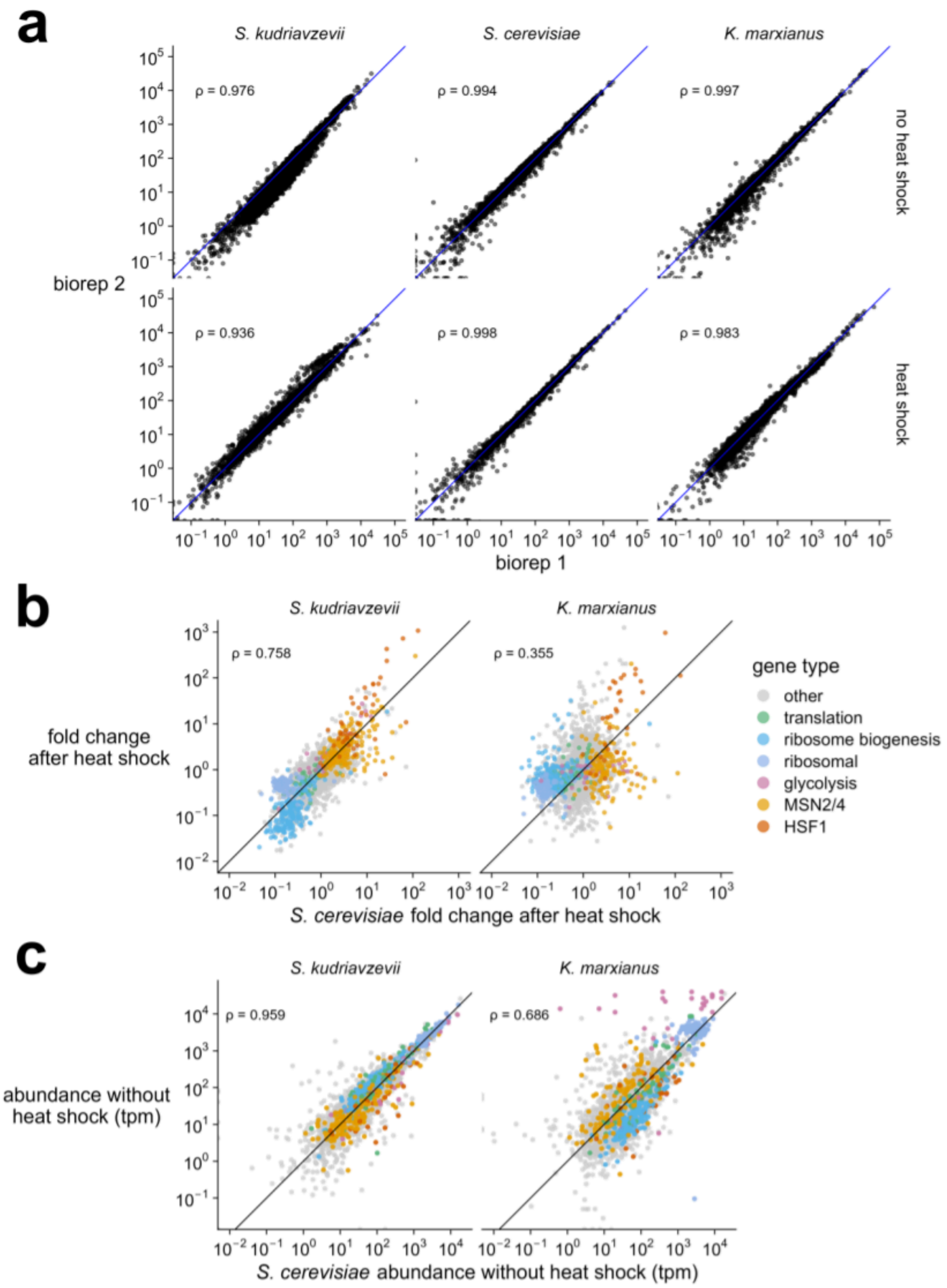

Figure S3. Strong correlations in biological replicates and within treatment comparisons.
a Transcript abundance (transcripts per million, tpm) between biological replicates in each species. Correlations are represented by Pearson's rho (ρ) calculated between replicates. **b** Fold change distribution for groups of genes (colored by gene type) after stress in each species. Correlations are represented by Pearson's rho (ρ) calculated between species. **c** Transcript abundance (transcripts per million, tpm) in each species without a heat shock. Correlations are represented by Pearson's rho (ρ) calculated between species.

Supplementary Figure S4.

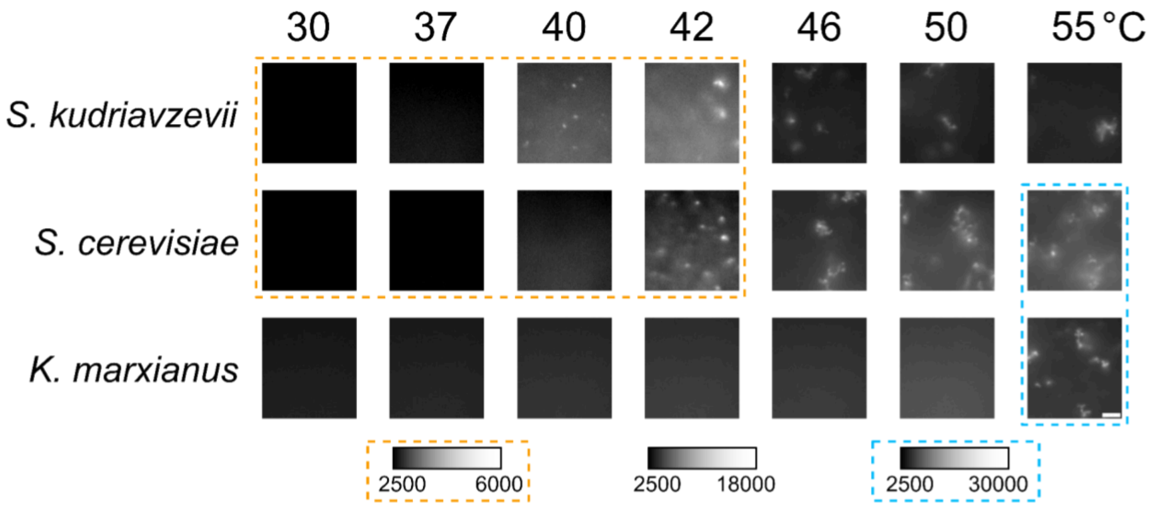

Figure S4: Microscopic visualization of condensation at increasing temperatures. Wide-field fluorescence imaging of Pab1 condensate formation at species-specific temperatures. Fluorescently labeled Pab1 (10 μM, 2% labeled) from each of three fungal species was held at 30°C for four minutes, then incrementally heated to higher temperatures for four minutes each. Gray scales indicate brightness in arbitrary units (AU). Three sets of brightness scalings were required to visualize condensates across these different temperatures; images employing scalings other than 2,500–18,000 (AU) are indicated by dotted-line groupings (orange, blue). Scale bar (bottom right, *K. marxianus* 55°C), 5 μm.

Supplementary Figure S5.

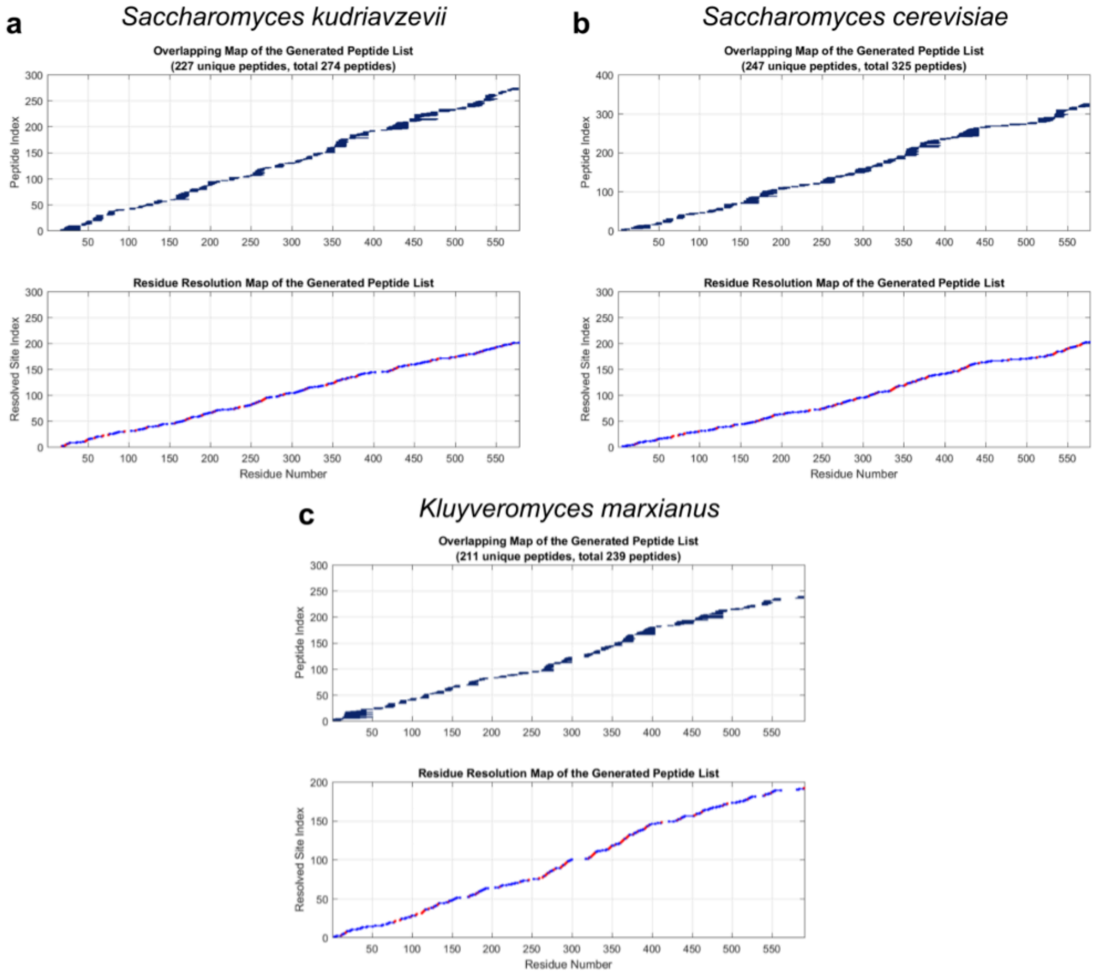

Figure S5: **Peptide maps for hydrogen-deuterium exchange studies of Pab1 orthologs from three budding yeast species**.
a *Saccharomyces kudriavzevii*, a cryophile. **b** *Saccharomyces cerevisiae*, a mesophile. **c** *Kluyveromyces marxianus*, a thermotolerant species.

Supplementary Table S1.

Well	Protein	Baseline radius (nm)	Baseline radius SD (nm)	SD check
F2	SkPab1WT	4.91	0.10	TRUE
H3	KmPab1MV.I	5.02	0.09	TRUE
K4	KmPab1WT	4.87	0.15	TRUE
K5	KmPab1MV.A	4.75	0.09	TRUE
K9	SkPab1MV.A	4.75	0.09	TRUE
L12	SkPab1MV.I	5.17	0.23	TRUE
NA	J-ScPab1MV.A	4.31	0.08	TRUE
NA	J-ScPab1MV.I	4.35	0.05	TRUE
NA	J-ScPab1WT	4.39	0.08	TRUE

Table 1. Pab1 baseline size estimations. Well indicates DLS experimental well; Protein indicates species abbreviation + Pab1 + mutant version; baseline_radius shows value of the mean radius in nm of measurements below 35°C; Baseline radius SD indicates the standard deviation of the baseline radius under 35°C; SD check: TRUE if baseline_radius_sd is less than 5% of baseline_radius. Data for *S. cerevisiae* are from (Riback et al. 2017).

Supplementary Table S2.

Well	Protein	T_{demix}	Radius	Rep	Species	Mutant
F2	SkPab1WT	38.1963	10.4914	rep_1	Skud	WT
H3	KmPab1MV.I	46.0497	9.03576	rep_1	Kmarx	MV.I
K4	KmPab1WT	48.9093	10.2584	rep_4	Kmarx	WT
K5	KmPab1MV.A	50.8701	9.70903	rep_2	Kmarx	MV.A
K9	SkPab1MV.A	39.6627	11.0515	rep_2	Skud	MV.A
L12	SkPab1MV.I	36.5503	9.43427	rep_4	Skud	MV.I
NA	J-ScPab1MV.A	40.7226	9.13283	rep_1	Scere	WT
NA	J-ScPab1MV.I	39.2875	8.23502	rep_1	Scere	MV.I
NA	J-ScPab1WT	42.7316	8.99349	rep_1	Scere	MV.A

Table 2. Pab1 $T_{condense}$ and estimations and size measurements. Well indicates DLS experimental well; Protein indicates species abbreviation + Pab1 + mutant version; T_{demix} represents the temperature where the radius is as close to double the average baseline value below 35°C; Radius is the measured diameter in nm of the radius and T_{demix} temperature; rep indicates which replicate is used for the calculation; Species shows an abbreviation of species used; Mutant indicates the version of Pab1 mutant used. Data for *S. cerevisiae* are from (Riback et al. 2017).

References

Riback, Joshua A., Christopher D. Katanski, Jamie L. Kear-Scott, Evgeny V. Pilipenko, Alexandra E. Rojek, Tobin R. Sosnick, and D. Allan Drummond. 2017. “Stress-Triggered Phase Separation Is an Adaptive, Evolutionarily Tuned Response.” *Cell* 168 (6): 1028–40.e19.
