# Supplementary material for: An adaptive biomolecular condensation response is conserved across environmentally divergent species
Source: Nat Commun. 2024 Apr 11;15:3127. doi: 10.1038/s41467-024-47355-9 (PMC11009240; doi:10.1038/s41467-024-47355-9)
Supplement: Supplementary file 3 — Reporting Summary [file 41467_2024_47355_MOESM3_ESM.pdf]

nature portfolio

Corresponding author(s): D. Allan Drummond

Last updated by author(s): 3 March 2024

Reporting Summary

Nature Portfolio wishes to improve the reproducibility of the work that we publish. This form provides structure for consistency and transparency in reporting. For further information on Nature Portfolio policies, see our Editorial Policies and the Editorial Policy Checklist.

Statistics

For all statistical analyses, confirm that the following items are present in the figure legend, table legend, main text, or Methods section.

n/a	Confirmed	
☐	☒	The exact sample size (*n*) for each experimental group/condition, given as a discrete number and unit of measurement
☐	☒	A statement on whether measurements were taken from distinct samples or whether the same sample was measured repeatedly
☒	☐	The statistical test(s) used AND whether they are one- or two-sided<br>*Only common tests should be described solely by name; describe more complex techniques in the Methods section.*
☒	☐	A description of all covariates tested
☒	☐	A description of any assumptions or corrections, such as tests of normality and adjustment for multiple comparisons
☐	☒	A full description of the statistical parameters including central tendency (e.g. means) or other basic estimates (e.g. regression coefficient) AND variation (e.g. standard deviation) or associated estimates of uncertainty (e.g. confidence intervals)
☒	☐	For null hypothesis testing, the test statistic (e.g. *F*, *t*, *r*) with confidence intervals, effect sizes, degrees of freedom and *P* value noted<br>*Give P values as exact values whenever suitable.*
☒	☐	For Bayesian analysis, information on the choice of priors and Markov chain Monte Carlo settings
☒	☐	For hierarchical and complex designs, identification of the appropriate level for tests and full reporting of outcomes
☐	☒	Estimates of effect sizes (e.g. Cohen's *d*, Pearson's *r*), indicating how they were calculated

Our web collection on statistics for biologists contains articles on many of the points above.

Software and code

Policy information about availability of computer code

Data collection	No commercial, open source, or custom code was used to collect data.
Data analysis	Code to reproduce analyses is included in the Source Data file.

For manuscripts utilizing custom algorithms or software that are central to the research but not yet described in published literature, software must be made available to editors and reviewers. We strongly encourage code deposition in a community repository (e.g. GitHub). See the Nature Portfolio guidelines for submitting code & software for further information.

Data

Policy information about availability of data

All manuscripts must include a data availability statement. This statement should provide the following information, where applicable:

- Accession codes, unique identifiers, or web links for publicly available datasets
- A description of any restrictions on data availability
- For clinical datasets or third party data, please ensure that the statement adheres to our policy

The yeast proteome is available from the Saccharomyces Genome Database (http://sgd-archive.yeastgenome.org/S288C_reference/orf_protein/. Sequencing data have been deposited in GEO under accession code GSE234499. Mass spectrometry proteomics data have been deposited to the ProteomeXchange Consortium via the PRIDE (108) partner repository with the dataset identifiers PXD044702 and PXD044970. Raw and processed flow cytometry data, HDX-MS data, growth data,

TSP-LC-MS/MS data, and DLS data to reproduce all figures have been deposited to Dryad (https://doi.org/10.5061/dryad.w3r2280w6) or GitHub (https://github.com/drummondlab/conservation-of-conservation-2024).

Research involving human participants, their data, or biological material

Policy information about studies with human participants or human data. See also policy information about sex, gender (identity/presentation), and sexual orientation and race, ethnicity and racism.

Reporting on sex and gender	NA
Reporting on race, ethnicity, or other socially relevant groupings	NA
Population characteristics	NA
Recruitment	NA
Ethics oversight	NA

Note that full information on the approval of the study protocol must also be provided in the manuscript.

Field-specific reporting

Please select the one below that is the best fit for your research. If you are not sure, read the appropriate sections before making your selection.

☒ Life sciences ☐ Behavioural & social sciences ☐ Ecological, evolutionary & environmental sciences

For a reference copy of the document with all sections, see nature.com/documents/nr-reporting-summary-flat.pdf

Life sciences study design

All studies must disclose on these points even when the disclosure is negative.

Sample size	1. For growth curve data, at least two biological replicates for each species at each temperature were used to calculate maximum specific growth rate at that temperature, producing growth data which were fit with the cardinal temperature model with inflection. This sample size is sufficient to demonstrate reproducibility.<br>2. DLS was performed on each purified protein sample at least three times (specific n reported in the caption), and representative samples were chosen for presentation. This sample size is sufficient to demonstrate reproducibility.<br>3. Flow cytometry data represent size-normalized medians >5,000 cells at each temperature, with distributional variance depicted in Figure S2A. This sample size is sufficient to demonstrate reproducibility.
Data exclusions	In flow cytometry, subpopulations of cells that exhibited high autofluorescence (abnormally high BL2 signal, indicative of cell death) were removed from the analysis; at least 5,000 cells per sample remained. The same parameters were used for each yeast strain for cell death removal where applicable, and analysis of filtering is available in the Dryad repository: https://doi.org/10.5061/dryad.w3r2280w6
Replication	All attempts at replication were successful and gave similar results.<br>1. Rna sequencing of biological replicates reflect high reproducibility at both heat shock and non-heat-shock temperatures (Pearson correlation > 0.93, Figure S3A).<br>2. Growth data were collected from at least two biological replicates for each species and temperature.<br>3. Two spot assays were performed on the Pab1 in vivo mutants (Figure 4E, S1B).
Randomization	Samples were run on corresponding instruments in a randomized order for relevant experiments (flow cytometry, DLS). Samples that were to be directly compared were collected from the same starting culture to avoid potential batch effects (mass spectrometry, RNA-seq, flow cytometry, spot assays).
Blinding	The investigators were not blinded during data collection due t osampling and treatment protocols. Computational analysis was not blinded as the extra layer of processing was judged to be unnecessary given the large effect sizes.

Reporting for specific materials, systems and methods

We require information from authors about some types of materials, experimental systems and methods used in many studies. Here, indicate whether each material, system or method listed is relevant to your study. If you are not sure if a list item applies to your research, read the appropriate section before selecting a response.

Materials & experimental systems

n/a	Involved in the study
☒	☐ Antibodies
☒	☐ Eukaryotic cell lines
☒	☐ Palaeontology and archaeology
☒	☐ Animals and other organisms
☒	☐ Clinical data
☒	☐ Dual use research of concern
☒	☐ Plants

Methods

n/a	Involved in the study
☒	☐ ChIP-seq
☐	☒ Flow cytometry
☒	☐ MRI-based neuroimaging

Plants

Seed stocks	NA
Novel plant genotypes	NA
Authentication	NA

Flow Cytometry

Plots

Confirm that:

☐ The axis labels state the marker and fluorochrome used (e.g. CD4-FITC).

☐ The axis scales are clearly visible. Include numbers along axes only for bottom left plot of group (a 'group' is an analysis of identical markers).

☐ All plots are contour plots with outliers or pseudocolor plots.

☐ A numerical value for number of cells or percentage (with statistics) is provided.

Methodology

Sample preparation	Cells were grown overnight to OD600 ~0.05 in SC-complete medium with 2% dextrose, temperature-treated for 20 minutes, and allowed to recover for 3 hours with shaking at 23°C.
Instrument	Data were collected on the AttuneNxT Acoustic Focusing Cytometer (Thermo Fisher) at 100 uL/min. At least 20,000 events were recorded per 100 uL of cells. Voltage was set as follows: Forward scatter: 1; Side scatter: 200; YL2 (mCherry): 540; VL1 (autofluoresscence): 400.
Software	Data were collected on the Attune NxT Flow Cytometer software, and were analyzed in R version 4.2.2. Custom analysis scripts are available in the Dryad repository: https://doi.org/10.5061/dryad.w3r2280w6
Cell population abundance	At least 20,000 events were recorded per 100 uL of cells.
Gating strategy	All experiments were performed with the same voltage set, and the fluorescence values reported reflect forward scatter area and autofluorescence-normalized values. Subpopulations of cells that exhibited high autofluorescence (abnormally high BL2 signal) were removed from the analysis; at least 5,000 cells per sample remained. The same parameters were used for each yeast strain for cell death removal where applicable, and analysis of filtering is available in the Dryad repository: https://doi.org/10.5061/dryad.w3r2280w6. Fold-changed values were calculated from mock-treated cells grown at 23°C.

☒ Tick this box to confirm that a figure exemplifying the gating strategy is provided in the Supplementary Information.
